# The landscape of immune microenvironment in lung adenocarcinoma and squamous cell carcinoma based on PD‐L1 expression and tumor‐infiltrating lymphocytes

**DOI:** 10.1002/cam4.2580

**Published:** 2019-10-11

**Authors:** Lu Chen, Mian‐Fu Cao, Xiang Zhang, Wei‐Qi Dang, Jing‐Fang Xiao, Qing Liu, Yu‐Huan Tan, Yao‐Yao Tan, Yuan‐Yuan Xu, Sen‐Lin Xu, Xiao‐Hong Yao, You‐Hong Cui, Xia Zhang, Xiu‐Wu Bian

**Affiliations:** ^1^ Institute of Pathology and Southwest Cancer Center Southwest Hospital Third Military Medical University (Army Medical University) Chongqing China; ^2^ Key Laboratory of Tumor Immunopathology Ministry of Education of China Chongqing China

**Keywords:** NSCLC, PD‐L1, TIL, TMIT

## Abstract

**Aims:**

The aim of this study was to investigate the tumor microenvironment immune types (TMIT) based on tumor cell programmed cell death ligand 1 (PD‐L1) expression and tumor‐infiltrating lymphocytes (TILs) distribution and whether distinct TMIT subtypes (TMIT I, PD‐L1^high^/TIL^high^; TMIT II, PD‐L1^low^/TIL^low^; TMIT III, PD‐L1^high^/TIL^low^; and TMIT IV, PD‐L1^low^/TIL^high^) differentially affect clinical outcomes of patients with lung adenocarcinoma (LAC) and squamous cell carcinoma (SCC).

**Methods and results:**

Immunohistochemistry (IHC) was applied to evaluate the expression of PD‐L1 and the spatial distribution of programmed cell death 1 (PD‐1) and CD8 TILs on the surgically resected specimens from 205 cases of LAC and 149 cases of SCC. PD‐1 and CD8 TILs were more frequently distributed in SCC than those in LAC, regardless of their infiltrating in the tumor islets or stroma. The density of TILs was a poor prognostic factor in LAC but a favorable one in SCC. PD‐L1 levels and its clinical prognostic significance differed in LAC vs SCC. LAC patients with TMIT III and SCC patients with TMIT I had the longest survival, respectively (*P* = .0197 and .0049). Moreover, TMIT stratification based on tumor cell PD‐L1 expression and stromal CD8^+^ TILs could be considered as an independent prognostic factor of SCC patients' survival as determined by both univariate and multivariate analysis.

**Conclusion:**

Our study indicates that different type of TMIT provides its specific microenvironment with diverse impact on survival of LAC and SCC patients and highlights the importance of the integrative assessment of PD‐L1 status and TILs' spatial distribution to predict patients' prognosis.

## Graphical abstract

Our study indicates that different type of TMIT provides its specific microenvironment with diverse impact on survival of lung adenocarcinoma and squamous cell carcinoma patients and highlights the importance of the integrative assessment of programmed cell death ligand 1 status and tumor‐infiltrating lymphocytes' spatial distribution to predict patients' prognosis.

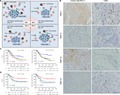

## 1 INTRODUCTION

Lung cancer is the leading cause of cancer mortality for human beings, although there are significant advances in therapeutic strategies.^1^ Pathologically, most of the lung cancer is non‐small cell lung cancer (NSCLC), mainly including lung adenocarcinoma (LAC) and squamous cell carcinoma (SCC).^2^ Recently, increasing evidence has shown that tumor cells express programmed cell death ligand 1 (PD‐L1) to escape T cells that express programmed cell death protein 1 (PD‐1), thereby promoting tumor progression.^3^ Programmed cell death protein 1/PD‐L1 immune checkpoint inhibitors (ICI) have thus been regarded as a promising therapeutic strategy for lung cancer.^4^, ^5^

The membrane expression of PD‐L1 by tumor cells affects the prognosis and immunotherapeutic effect.^6^, ^7^, ^8^ However, blocking therapy is also effective in some patients without PD‐L1 expression as pathologically determined on their tumor specimens.^9^, ^10^ Standardization of PD‐L1 detection for clinical application still remains arguable, although some controversies are partially attributed to antibody specificity and different evaluation methods.^11^, ^12^ Additionally, attempts to predict prognosis and determine whether cancer patients need ICI treatment through standardized PD‐L1 scoring have not considered the extend and intensity of PD‐L1 expression simultaneously. Importantly, tumor microenvironment is another critical factor that influences clinical outcomes and response to ICI.^13^, ^14^ Therefore, the combinations of biomarkers aimed at prognosis prediction and personalized therapy can be obtained by an extended analysis of tumor microenvironment immune types (TMIT). Specifically, according to the expression of PD‐L1 on tumor cells and tumor‐infiltrating lymphocytes (TILs), TMIT classification has been recently proposed by Teng et al,^15^ including TMIT I adaptive immune resistance (PD‐L1 positive and high TIL), TMIT II immune ignorance (PD‐L1 negative and low TIL), TMIT III intrinsic induction (PD‐L1 positive and low TIL), and TMIT IV immune tolerance (PD‐L1 negative and high TIL). Several studies based on database analysis have revealed that the proposed classification of TMIT subtypes is important to tailor optimal immunotherapeutic strategies.^16^, ^17^ Nevertheless, the pathological characterizations of TMIT based on both the expression of PD‐L1 on tumor cells and the spatial distribution of TIL especially in LAC and SCC remain unknown.

This study was performed to determine how TMIT classification based on PD‐L1 and TIL affect the prognostic prediction of the clinical outcome of patients with different histological subtyping of NSCLC. Surgically resected specimens from 205 cases of LAC and 149 cases of SCC were morphometrically analyzed to investigate PD‐L1 expression on tumor cells and immune cells and to determine the spatial distribution of PD‐1 and CD8 TILs.

## 2 MATERIALS AND METHODS

### 2.1 Patients

The study was approved by the institutional review board of Southwest Hospital, Third Military Medical University (Army Medical University), Chongqing, China (2013KY41). The tumor specimens of patients from 205 LAC and 149 SCC were included. All patients underwent surgical resection without previous therapy and pathologically diagnosed at Southwest Hospital from 2006 to 2012.

### 2.2 Immunohistochemistry staining analysis

Immunohistochemistry (IHC) staining of tissue sections (3‐µm thick) was applied using Envision IHC Detection System Kit (Code K5007; DAKO) and monoclonal antibodies against PD‐1 (D4W2J; Cell Signaling, Danvers), CD8 (D8A8Y; Cell Signaling), and PD‐L1 (E1L3N; Cell Signaling). For PD‐1 and CD8 TIL counting, five representative fields (magnification ×400) in the tumor islets, peritumoral stroma, invasive margin, and germinal center from each slide were randomly selected under an Olympus BX51 microscope (Olympus), respectively. Image‐Pro Plus 5.0 software (Media Cybernetics) was applied to measure the areas of each region and the corresponding number of nucleated PD‐1^+^ and CD8^+^ T cells. The data were expressed as cells/mm^2^.^18^ Data analysis was firstly performed on 20 samples and repeated 2 weeks later in order to ensure consistence, and good correlations were found for above two sets of TIL counting (0.994, 95% CI 0.984‐0.999; *P* < .0001) and for the data between automatic cell count and manual cell count (0.997, 95% CI 0.995‐0.999; *P* < .0001).

According to the current convention for PD‐L1 expression assessment, we scored the percentages of PD‐L1 membranous positivity on tumor cells without considering staining intensity. Different thresholds for PD‐L1 high expression were determined as ≥1%, ≥10%, or ≥50%. When grouping of PD‐L1 score was not concordant between two pathologists, consensus was made for discrepancies by reviewing the slide and scoring together. PD‐L1^+^ immune cells (cells/mm^2^) in the peritumoral stroma and invasive margin were analyzed using the aforementioned cell counting method. For comprehensive consideration of the intensity of tumor cell PD‐L1 staining, five representative fields (magnification ×200) in the region of tumor cells from each slide were randomly selected. Image‐Pro Plus 5.0 software was utilized to measure the areas of tumor cell region excluding stroma areas and the corresponding integrated optic density (IOD) of the tumor cells with PD‐L1 expression. The intensity of PD‐L1 staining was presented as IOD per unit area. Recognition of tumor cell regions was conducted under the guidance and confirmation of pathologists.

### 2.3 Statistics

Statistical analyses were done using SPSS 19.0 (SPSS Inc). The *t* test was used for comparative analysis. The optimal cutoff level was determined by receiver operating characteristics (ROC) curve.^19^ Briefly, in the ROC curves, *x*‐axis was plotted as “1‐specificity” (false positivity) and the y‐axis as “sensitivity” (true positivity). The optimal cutoff value was determined by the Youden index (*Y*), which was the point with maximum sensitivity and specificity (*Y* = sensitivity+specificity − 1). The diagonal line referred to ‘‘random guess.’’ If the Youden index demonstrated an asymptomatic significance above 0.2 or an inadequately shaped ROC curve, the median was selected for the cutoff value. Kaplan‐Meier method was conducted to examine survival. The correlation between data and clinical pathological parameters was determined by Pearson's Chi‐square (*χ^2^*) test. A multivariate Cox regression model was established to identify independent prognostic factors. *P* < .05 was regarded as statistical significance.

## 3 RESULTS

### 3.1 TIL distribution and its relation to survival

**Figure 1:**
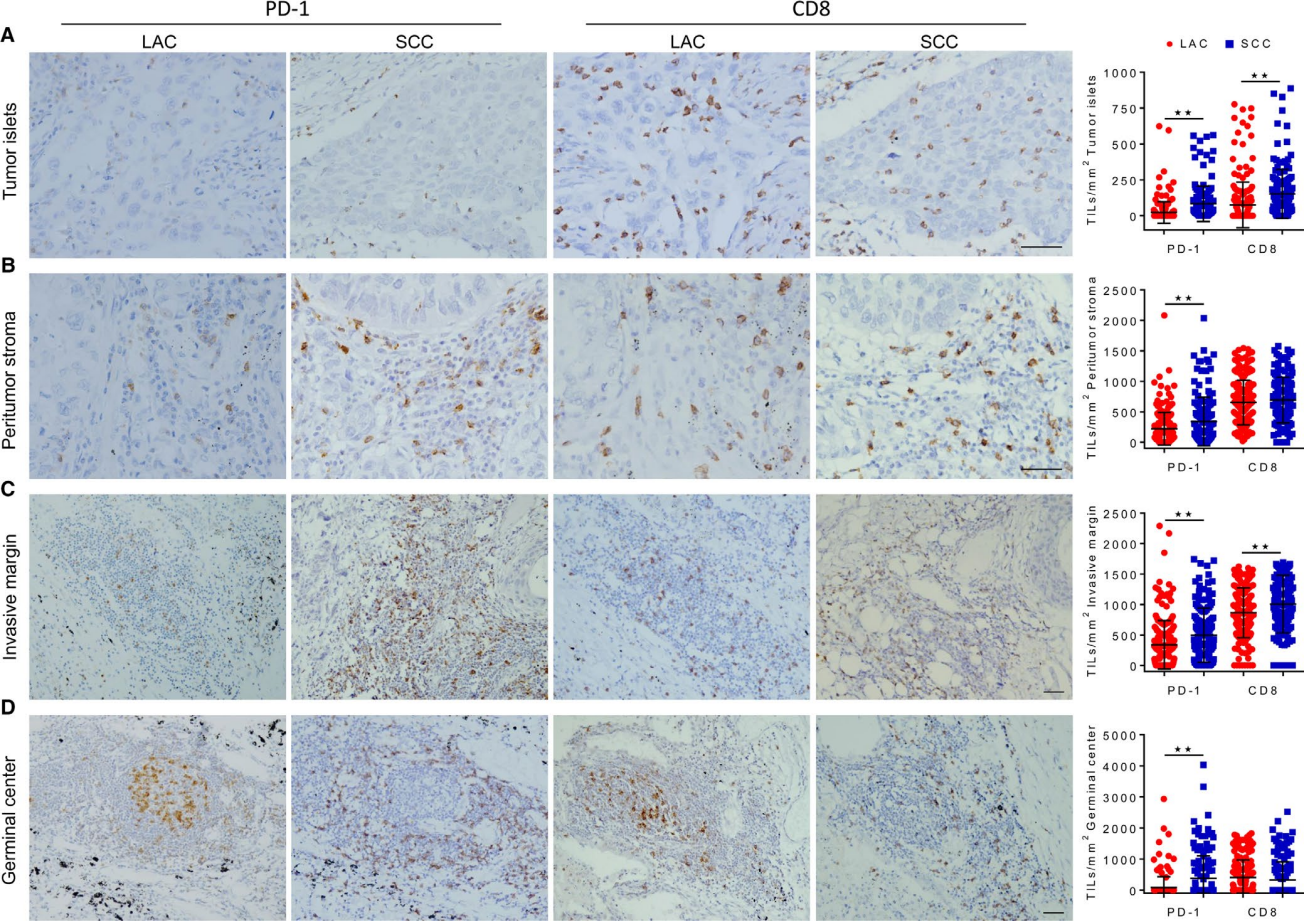
Distribution of programmed cell death 1 (PD‐1) and CD8 tumor‐infiltrating lymphocytes (TIL) in lung adenocarcinoma (LAC) and squamous cell carcinoma (SCC) samples. Representative images of immunohistochemical staining showing the distribution of PD‐1 and CD8 TIL, and dot plot diagrams comparing the density of PD‐1^+^ cells and CD8^+^ T cells in the tumor islets (A), peritumoral stroma (B), invasive margin (C), and germinal center (D) of LAC and SCC samples. **P < .01. *t* test. Scale bar, 50 µm

**Figure 2:**
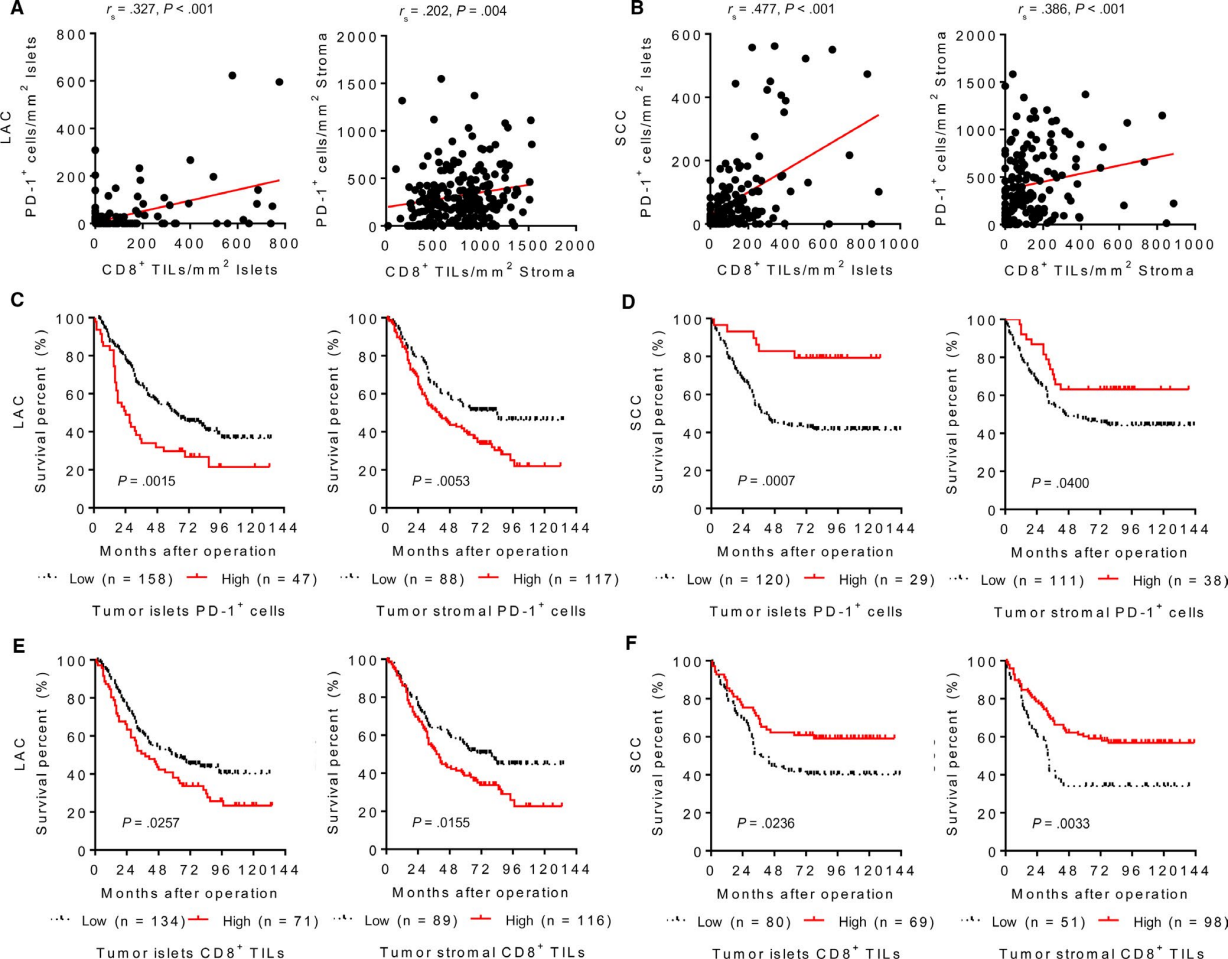
Kaplan‐Meier survival curves for the spatial distribution of programmed cell death 1 (PD‐1) and CD8 tumor‐infiltrating lymphocytes in lung adenocarcinoma (LAC) and squamous cell carcinoma (SCC). The correlation analysis between PD‐1^+^ cells and CD8^+^ T cells in the tumor islets and stroma of LAC (A) and SCC (B) samples. (C) LAC patients with a high density of PD‐1^+^ cells in the both tumor islets and stroma had a shorter survival. (D) SCC patients with a high density of PD‐1^+^ cells in the both tumor islets and stroma had a longer survival. High number of CD8^+^ T cells in the both tumor islets and stroma was associated with a poor prognosis in LAC patients (E), but with a better outcome in SCC patients (F). *P*‐values were calculated using the log‐rank test

The spatial distribution of PD‐1 and CD8 TILs was analyzed on the specimens from 205 cases of LAC and 149 cases of SCC by an IHC examination (Figure 1; Figure S1). Statistical results showed that the number of PD‐1 and CD8 TILs was significantly different between LAC and SCC samples, regardless of their infiltrating in the tumor islets (Figure 1A), peritumoral stroma (Figure 1B), invasive margin (Figure 1C), or germinal center (Figure 1D). Because the germinal center near the pulmonary alveoli belongs to a paracancerous component and both peritumoral stroma and invasive margin are tumor stromal component, only TILs in the tumor islets and stroma are classified and considered in the subsequent analysis of this study (Figure S2). Correlation analysis displayed that the spatial distribution of PD‐1^+^ cells was correlated with that of CD8^+^ T cells in both LAC and SCC samples (Figure 2A,B). As shown in Figure 2C, LAC patients carrying high density of PD‐1^+^ cells in both tumor islets (*P* = .0015) and stroma (*P* = .0053) had a shorter survival. Conversely, a better outcome was observed in SCC patients with high number of PD‐1^+^ cells infiltrating in the tumor islets (*P* = .0007) and stroma (*P* = .0400) (Figure 2D). Similarly, high accumulation of CD8^+^ T cells in the tumor islets and stroma was associated with a poor prognosis in LAC (*P* = .0257 and .0155, respectively), but with a favorable prognosis in SCC (*P* = .0236 and .0033, respectively) (Figure 2E,F). The correlations between TIL distribution and clinical parameters are shown in Table S1. Thus, PD‐1 and CD8 TIL are more frequently observed in SCC than those in LAC, and the clinical significance of TILs' spatial distribution is diverse between LAC and SCC.

### 3.2 PD‐L1 expression and its relation to survival

**Figure 3:**
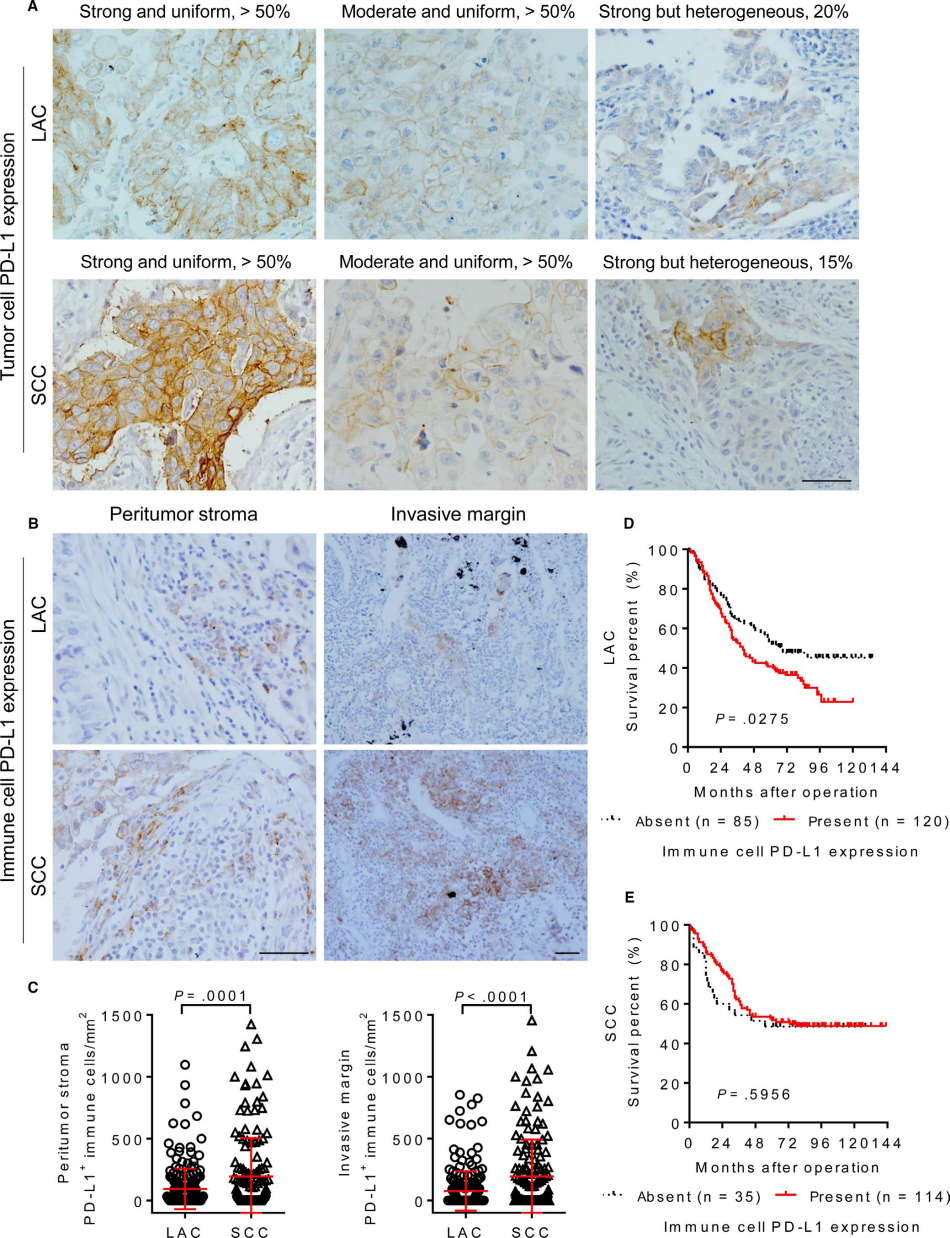
Programmed cell death ligand 1 (PD‐L1) expression in the tumor cell and immune cell. A, Representative images of immunohistochemical staining presenting a great inter‐patient variability of tumor cell PD‐L1 expression in different samples of lung adenocarcinoma (LAC) and squamous cell carcinoma (SCC). B, Representative images displaying PD‐L1^+^ immune cells in the peritumoral stroma and invasive margin of LAC and SCC samples. Scale bar, 50 µm. C, Dot plot diagrams comparing the density of PD‐L1^+^ immune cells in the peritumoral stroma and invasive margin between LAC and SCC samples. *t* test. D, The presence of PD‐L1^+^ immune cell was associated with poor prognosis of LAC patients. E, Immune cell PD‐L1 expression was not associated with SCC patients' survival. *P*‐values were calculated using the log‐rank test

**Figure 4:**
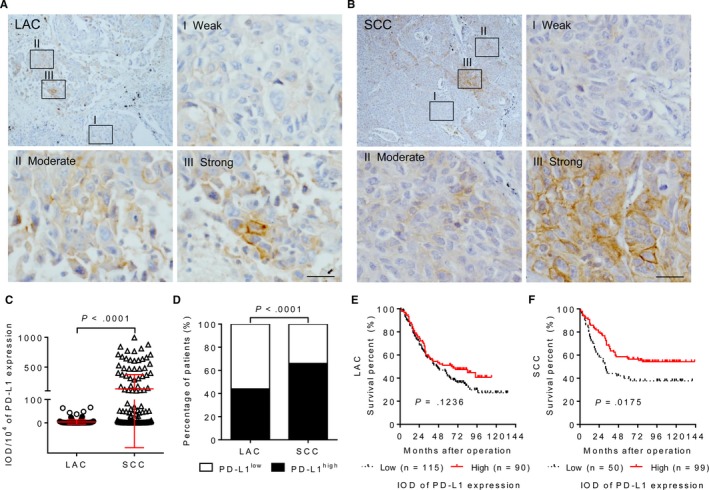
Geographic variability of the programmed cell death ligand 1 (PD‐L1) expression of tumor cell. Representative images of immunohistochemical staining exhibiting a great intra‐patient heterogeneity of PD‐L1 levels in one same tumor tissue of lung adenocarcinoma (LAC) (A) and squamous cell carcinoma (SCC) (B). Scale bar, 25 µm. C, Dot plot diagrams comparing the integrated optic density (IOD) value of PD‐L1 expression by tumor cells in LAC and SCC patients. *t* test. D, Histogram displaying frequency of high PD‐L1 expression by tumor cells in LAC and SCC patients. *χ* ^2^ test. E, Tumor cell PD‐L1 expression was not significantly associated with LAC patients' survival. F, SCC patients with high expression of PD‐L1 on tumor cells having a longer survival. *P*‐values were calculated using the log‐rank test

The assessment of PD‐L1 expression in the tumor cells (Figure 3A) and immune cells (Figure 3B) was then carried out. As compared with LAC, the positive staining of PD‐L1 in the both tumor cells and immune cells was more frequently detected in SCC (Table 1). Figure 3C showed that SCC patients had a higher number of PD‐L1^+^ immune cells infiltrating in the peritumoral stroma (*P* = .0001) and invasive margin (*P* < .0001). Moreover, the presence of PD‐L1^+^ immune cell was associated with a poor prognosis in LAC (*P* = .0275; Figure 3D), but not in SCC (*P* = .5956; Figure 3E). Beside for the variability of the percentage and intensity of PD‐L1 staining between different samples, such variability was also obvious in the different tumor areas on the same specimen derived from LAC (Figure 4A) and SCC (Figure 4B). In order to evaluate the expression of PD‐L1 more comprehensively, IOD score based on the proportion and intensity of tumor cell PD‐L1 positivity was then used for semiquantitative analysis. The results showed that SCC displayed higher PD‐L1 IOD value as compared with LAC (Figure 4C,D). Associations between the percentage of PD‐L1 positivity and the survivals of LAC and SCC patients were not obvious (Figure S3). Similarly, there was no significant correlation between survival and PD‐L1 IOD value in LAC (*P* = .1236; Figure 4E), while SCC patients with high PD‐L1 expression had a longer survival (*P* = .0175; Figure 4F). The correlations between PD‐L1 expression and clinicopathological characteristics are summarized in Table S2. These results indicate the heterogeneity of PD‐L1 expression and the difference of its prognostic value among different histological subtypes of lung cancer.

**Table 1:** The difference of PD‐L1 expression between lung adenocarcinoma and squamous cell carcinoma

	Case	Tumor cell PD‐L1 expression						Immune cell PD‐L1 expression			
		PD‐L1^+^		PD‐L1^+^		PD‐L1^+^		Peritumoral stroma		Invasive margin	
		<1%	≥1%	<10%	≥10%	<50%	≥50%	Absent	Present	Absent	Present
LAC	205	152 (74.1)	53 (25.9)^a^	162 (79.0)	43 (21.0)^b^	180 (87.8)	25 (12.2)^c^	96 (46.8)	109 (53.2)^d^	125 (60.9)	80 (39.1)^e^
SCC	149	66 (44.3)	83 (55.7)	88 (59.1)	61 (40.9)	104 (69.8)	45 (30.2)	53 (35.6)	96 (64.4)	62 (41.6)	87 (58.4)

Note

Data shown as n (%).

Abbreviations: LAC, lung adenocarcinoma; PD‐L1, programmed cell death ligand 1; SCC, squamous cell carcinoma.

a *p* < .0001,

b *p* < .0001,

c *p* < .0001,

d *p* = .0385,

e *p* = .0004, *χ* ^2^ or Fisher's exact test.

### 3.3 The landscape of immune microenvironment based on PD‐L1 and TILs

**Figure 5:**
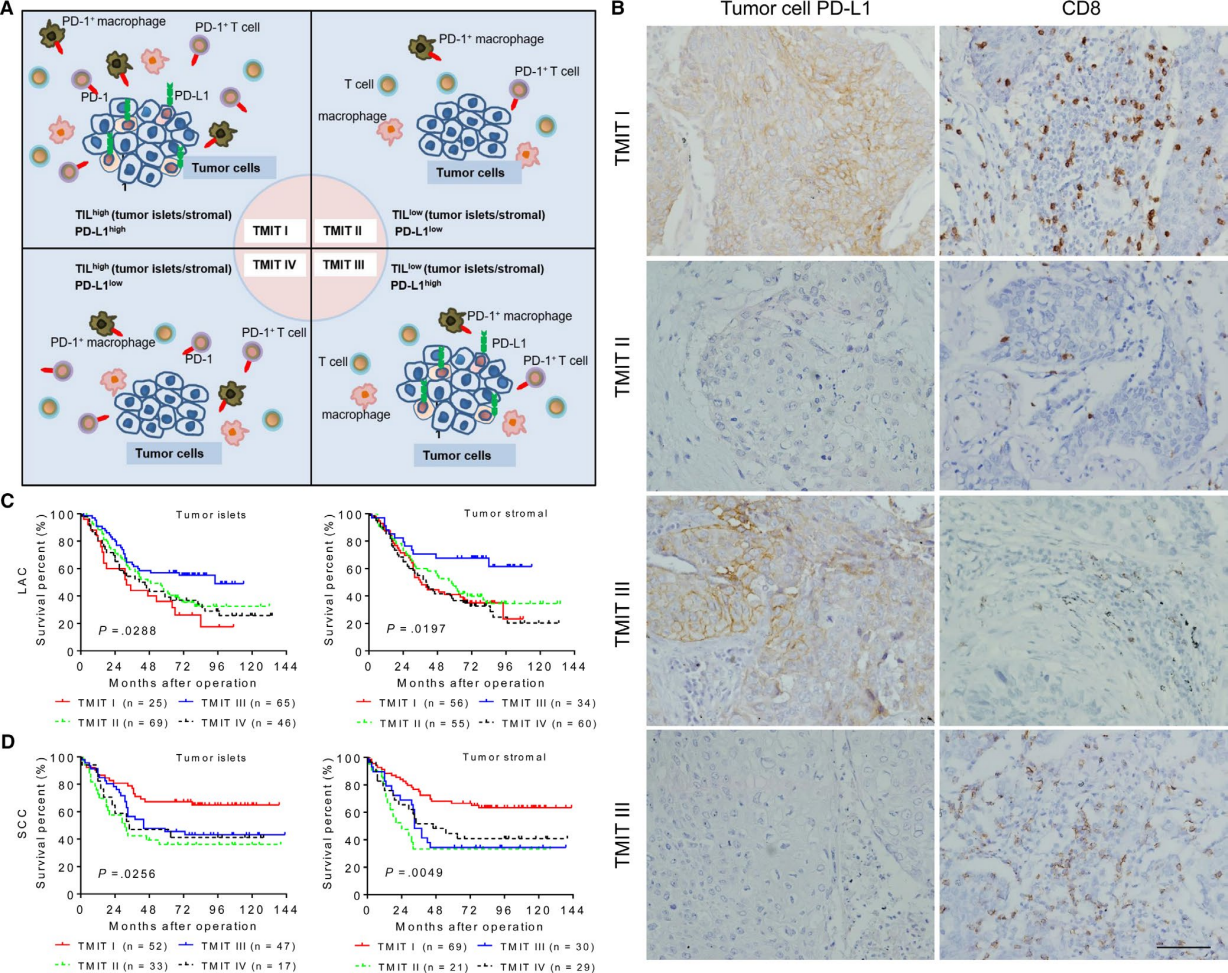
Kaplan‐Meier survival curves for tumor microenvironment immune types (TMIT) classification in lung adenocarcinoma (LAC) and squamous cell carcinoma (SCC) samples. A, Schematic presentation of analysis of TMIT based on tumor cell programmed cell death ligand 1 (PD‐L1) expression and tumor‐infiltrating lymphocytes (TIL) infiltrating in the tumor islets or stroma. B, Representative images of immunohistochemical staining showing four types of TMIT in SCC: TMIT I (PD‐L1^high^ and CD8^+^ TIL^high^), TMIT II (PD‐L1^low^ and CD8^+^ TIL^low^), TMIT III (PD‐L1^high^ and CD8^+^ TIL^low^), and TMIT IV (PD‐L1^low^ and CD8^+^ TIL^high^). LAC patients with TMIT III (C) and SCC patients with TMIT I (D) having the longest survival, respectively. *P*‐values were calculated using the log‐rank test

Based on the method proposed by Teng et al,^15^ we first carried out a stratification of TMIT according to the mRNA expression of PD‐L1 and CD8A from 385 cases of LAC and 351 cases of SCC obtained from The Cancer Genome Atlas (http://tcga-data.nci.nih.gov/tcga/) (Figure S4). The database analysis revealed that TIMT did not show a statistically significant predictive effect on survival (Table S3). We then performed a more detailed analysis of TMIT according to the IOD values of PD‐L1 together with the spatial distribution of CD8^+^ TILs (Figure 5A,B). Whether classified by tumor islets (*P* < .0001) or stromal (*P* = .0002) CD8^+^ T cells, the proportions of TMIT between LAC and SCC samples were different (Table 2). Additionally, the impact on clinical prognosis of such detailed classification of TMIT was apparent. In the case of high PD‐L1 IOD value, LAC patients with low density of TIL (TMIT III) in the tumor islets (*P* = .0288; Figure 5C left) and stroma (*P* = .0197; Figure 5C right) had the longest survival. Whether classified by tumor islets or stromal TIL, SCC patients with TMIT I adaptive immune resistance had the longest survival (*P* = .0256 and .0049, respectively), while the prognosis of TMIT II immune ignorance was poor (Figure 5D). Therefore, a comprehensive assessment of PD‐L1 expression and the spatial distribution of TIL allow us to comprehensively recognize TMIT characteristics and will affect patients' prognosis. Clinicopathologic correlations of TMIT are summarized in Table S4. The Cox regression analysis showed that TMIT classification based on PD‐L1 expression and stromal TIL could be considered as an independent prognostic factor in SCC as determined by both univariate and multivariate analysis (Table 3). These results demonstrate the difference of TMIT stratification and their significant prognostic value in LAC and SCC patients.

**Table 2:** The proportions of TMIT based on PD‐L1 expression and CD8^+^ tumor‐infiltrating lymphocytes between lung adenocarcinoma and squamous cell carcinoma

TMIT	LAC n (%)	SCC n (%)	*P*
Tumor cell PD‐L1 and TILs tumor islets			<.0001
TMIT I (PD‐L1^high^ and TIL^high^)	25 (12)	52 (35)	
TMIT II (PD‐L1^low^ and TIL^low^)	69 (34)	33 (22)	
TMIT III (PD‐L1^high^ and TIL^low^)	65 (32)	47 (32)	
TMIT IV (PD‐L1^low^ and TIL^high^)	46 (22)	17 (11)	
Tumor cell PD‐L1 and TILs tumor stromal			.0002
TMIT I (PD‐L1^high^ and TIL^high^)	56 (27)	69 (46)	
TMIT II (PD‐L1^low^ and TIL^low^)	55 (27)	21 (14)	
TMIT III (PD‐L1^high^ and TIL^low^)	34 (17)	30 (20)	
TMIT IV (PD‐L1^low^ and TIL^high^)	60 (29)	29 (20)	

Abbreviations: LAC, lung adenocarcinoma; PD‐L1, programmed cell death ligand 1; SCC, squamous cell carcinoma; TIL, tumor‐infiltrating lymphocytes; TMIT, tumor microenvironment immune types.

**Table 3:** Significant independent prognostic factors by Cox regression analysis

Factor	LAC (n = 205)				SCC (n = 149)			
	Univariate Cox regression		Multivariate Cox regression^a^		Univariate Cox regression		Multivariate Cox regression^a^	
	HR (95% CI)	*P*	HR (95% CI)	*P*	HR (95% CI)	*P*	HR (95% CI)	*P*
Gender								
Female	1.000				1.000			
Male	1.245 (0.872‐1.778)	.228			0.815 (0.200‐3.320)	.775		
Age								
≤60	1.000				1.000			
>60	0.967 (0.681‐1.372)	.849			1.282 (0.809‐2.032)	.290		
Smoking								
No	1.000				1.000			
Yes	0.918 (0.646‐1.306)	.636			0.806 (0.425‐1.528)	.509		
Stage								
I and II	1.000		1.000		1.000		1.000	
III and IV	3.831 (2.624‐5.594)	<.001	3.478 (2.333‐5.212)	<.001	2.251 (1.404‐3.608)	.001	2.062 (1.278‐3.329)	.003
Differentiation								
Well	1.000				1.000			
Moderate and poor	2.305 (1.410‐3.770)	.001			1.266 (0.511‐3.137)	.611		
Histology subtype								
Lepidic	1.000		1.000		—			
Acinar	2.419 (1.282‐4.563)	.006	2.026 (1.064‐3.859)	.032	—			
Papillary	1.989 (0.824‐4.803)	.126	1.738 (0.713‐4.238)	.224	—			
Solid	4.387 (2.189‐8.791)	<.001	2.873 (1.398‐5.908)	.004	—			
Tumor cell PD‐L1	0.998 (0.965‐1.011)	.297			1.000 (0.999‐1.001)	.480		
Immune cell PD‐L1	1.001 (1.000‐1.002)	.033			0.999 (0.999‐1.000)	.224		
PD‐1 tumor islets	1.004 (1.002‐1.006)	<.001	1.003 (1.001‐1.006)	.004	0.997 (0.994‐0.999)	.014		
PD‐1 tumor stromal	1.000 (1.000‐1.001)	.086			0.999 (0.999‐1.000)	.102		
CD8 tumor islets	1.001 (1.000‐1.002)	.012			0.998 (0.997‐1.000)	.062		
CD8 tumor stromal	1.001 (1.000‐1.001)	.012			0.999 (0.999‐1.000)	.061		
TMIT subtype								
Tumor cell PD‐L1 and TIL total								
TMIT I	1.000				1.000			
TMIT II	0.838 (0.522‐1.345)	.464			3.094 (1.667‐5.705)	.001		
TMIT III	0.394 (0.208‐0.744)	.004			2.073 (1.150‐3.736)	.015		
TMIT IV	1.029 (0.663‐1.596)	.899			1.587 (0.812‐3.104)	.177		
Tumor cell PD‐L1 and TIL tumor islets								
TMIT I	1.000				1.000			
TMIT II	0.728 (0.425‐1.244)	.246			2.531 (1.346‐4.761)	.004		
TMIT III	0.454 (0.255‐0.808)	.007			1.908 (1.050‐3.467)	.034		
TMIT IV	0.813 (0.461‐1.432)	.473			2.107 (0.972‐4.570)	.059		
Tumor cell PD‐L1 and TIL tumor stromal								
TMIT I	1.000				1.000		1.000	
TMIT II	0.865 (0.544‐1.374)	.538			2.784 (1.442‐5.376)	.002	2.671 (1.377‐5.181)	.004
TMIT III	0.413 (0.215‐0.794)	.008			2.365 (1.311‐4.268)	.004	2.155 (1.188‐3.909)	.012
TMIT IV	1.100 (0.709‐1.708)	.671			1.996 (1.077‐3.700)	.028	1.987 (1.072‐3.684)	.029

Note

Tumor cell PD‐L1, immune cell PD‐L1, PD‐1 tumor islets, PD‐1 tumor stromal, CD8 tumor islets, CD8 tumor stromal were continue variables.

Abbreviations: CI, confidence interval; HR, hazard ratio; LAC, lung adenocarcinoma; PD‐1, programmed cell death 1; PD‐L1, programmed cell death ligand 1; SCC, squamous cell carcinoma; TIL, tumor‐infiltrating lymphocytes; TMIT, tumor microenvironment immune types; TMIT I, PD‐L1^high^ and TIL^high^; TMIT II, PD‐L1^low^ and TIL^low^; TMIT III, PD‐L1^high^ and TIL^low^; TMIT IV, PD‐L1^low^ and TIL^high^.

a Only statistically significant variables obtained from the univariate model were included in the multivariate analysis. Results of multivariate analysis showing significant independent prognostic factors.

## 4 DISCUSSION

Programmed cell death 1/PD‐L1 ICI has shown promising results in patients with NSCLC.^20^, ^21^ Apart from clinicopathologic parameters and mutation‐defined molecular phenotypes,^22^ distinct TMIT is another key impact of clinical prognosis and therapy in lung cancer.^23^ The rational selection of personalized ICI therapy requires a deeper understanding of TMIT based on PD‐L1 and TIL. Meanwhile, the TMIT‐oriented research will provide important insights into the pathogenesis, progression, and treatment strategy of LAC and SCC. In order to evaluate the prognostic impact of different TMIT based on the geographic distribution of TILs and the level of PD‐L1 expression, we performed a retrospective systematic analysis of specimens from 205 LAC and 149 SCC patients. Our study indicates that different type of TMIT provides its specific microenvironment with diverse impact on survival of LAC and SCC patients.

Database analysis showed that TMIT was significantly associated with somatic mutations, neoantigen, PD‐L1 amplification, and oncogenic viral infection,^16^ but it was a comprehensive analysis of all types of tumors. Different lung cancer subtypes have a different biologic background and tissue characteristics.^24^ Subsequent genomic analysis indicated that TMIT was associated with mutation and neoantigen numbers in LAC but not in SCC.^17^ Based on the IHC analysis, some study reported that PD‐L1 expression was related to a longer^25^ or a shorter^26^, ^27^ survival of NSCLC patients, or was even not correlated with their survival.^28^ In this respect, tumor cell PD‐L1 IOD value was associated with prolonged survival in SCC, but immune cell expression was correlated with poor prognosis in LAC. The inter‐patient and inter‐tissue variability of PD‐L1 by our IHC detection demonstrates that the expression of PD‐L1 may differ between different lung cancer cohorts, and may be influenced by various factors, including tumor types, genetic backgrounds, and inflammation. It was reported that TMIT stratification of colorectal cancer was associated with high levels of microsatellite instability and neoantigen load, supporting better response to ICI.^29^ However, the analysis was based on the TILs counting in both tumor and adjacent stroma together. The exact microanatomic localization of TILs could be a key parameter in TMIT analysis of lung cancer. In our study, we paid attention to counting PD‐1 and CD8 TILs within tumor islet and stroma and found that the density of TILs was associated with a poor prognosis in LAC but conversely with a favorable one in SCC. Increased TILs have been reported to be a favorable prognostic factor in NSCLC.^30^ However, CD8^+^ T cells have been associated with a better^27^ or a worse^31^ prognosis in NSCLC. It may be due to different histological subtypes of NSCLC and whether CD8^+^ T cells are sufficiently activated.

The simultaneous assessment of tumor cells and immune ones allowed us to identify TMIT with differential impact on clinical outcome. Although our study showed that the clinical significance of TMIT analysis based on RNA data was not obvious, TMIT classification by an IHC analysis, especially according to the distribution of CD8^+^ T cells in the tumor islets or stroma, allowed us to identify TMIT with differential impact on clinical outcome. Specifically, we documented that LAC patients with TMIT III and SCC patients with TMIT I had the longest survival, respectively. TMIT stratification, especially based on the stromal CD8^+^ TILs, was a potential independent prognostic variable in SCC. Thus, the reservoir of stromal CD8^+^ TILs and the high expression of PD‐L1 resulted in longer survival in SCC patients, associated with the recent proposals that TMIT I patients are most likely to response to checkpoint blockade.^15^ However, this observation was found only in SCC, but not in LAC. Intriguingly, LAC patients with an accumulation of TILs, especially TMIT IV, had a shorter survival, indicating that CD8^+^ TILs may be partially activated in the immunosuppressive microenvironment of LAC. Moreover, the low expression of PD‐L1 in LAC might make the tumor microenvironment more attractive to inflammatory cells.^14^, ^28^ Accordingly, TMIT IV can lead to the heterogeneity of immune cells in distribution and phenotype. Conversely, inflammatory background may further upregulate the levels of PD‐L1 by inducing the secretion of PD‐L1 stimulator, such as interferon γ.^32^ Finally, inducible expression of PD‐L1 can be applied to potentially predict the response to ICI. These findings indicated that the stromal PD‐1 and CD8 TILs may have dual activating and inhibitory functions in different subtypes of NSCLC. It has been shown that PD‐1 is mainly expressed on CD8^+^ rather than CD4^+^ T cells,^33^, ^34^ and low PD‐1 expression in CD8^+^ T cells confers a survival advantage in NSCLC.^28^ In a study of mouse‐transplanted colon cancer, macrophages, apart from lymphocytes, expressed high level of PD‐1 to inhibit phagocytosis and tumor immunity.^35^ Our study underlines the need to further identify which immune cell subpopulation expresses PD‐1/PD‐L1 and its' predominant spatial distribution, and whether and to what extent it affects immune activation or inhibition.

Immunohistochemical detection of resected samples may not be entirely applicable to unresectable or advanced LAC or SCC cohorts, and future research will focus on its' correlation with biopsy samples. To strengthen the predictive value of TMIT, further analysis of patients treated with ICI is needed to determine whether ICI treatment significantly affects the spatial distribution of TILs. In conclusion, TMIT stratification is a potential prognostic factor in LAC and SCC. Our study suggests that an accurate pathological evaluation requires a comprehensive analysis of multiple variables within tumor microenvironment, and biomarker characterization based on such analysis enables us to predict patients' prognosis and possibly to better guide clinical treatment.

## AUTHOR CONTRIBUTIONS

X. Zhang and X.‐W. Bian contributed to study conception and design. S.‐L. Xu and L. Chen reviewed pathological slides. L. Chen analyzed the data and wrote manuscript draft. All authors contributed to data collection and manuscript editing.

## Data Availability

I confirm that my article contains a Data Availability Statement even if no data are available (list of sample statements) unless my article type does not require one. I confirm that I have included a citation for available data in my references section, unless my article type is exempt.
