# Supplementary material for: The landscape of immune microenvironment in lung adenocarcinoma and squamous cell carcinoma based on PD‐L1 expression and tumor‐infiltrating lymphocytes
Source: Cancer Med. 2019 Oct 11;8(17):7207–18. doi: 10.1002/cam4.2580 (PMC6885882; doi:10.1002/cam4.2580)
Supplement: Supplementary file 1 [file CAM4-8-7207-s001.pdf]

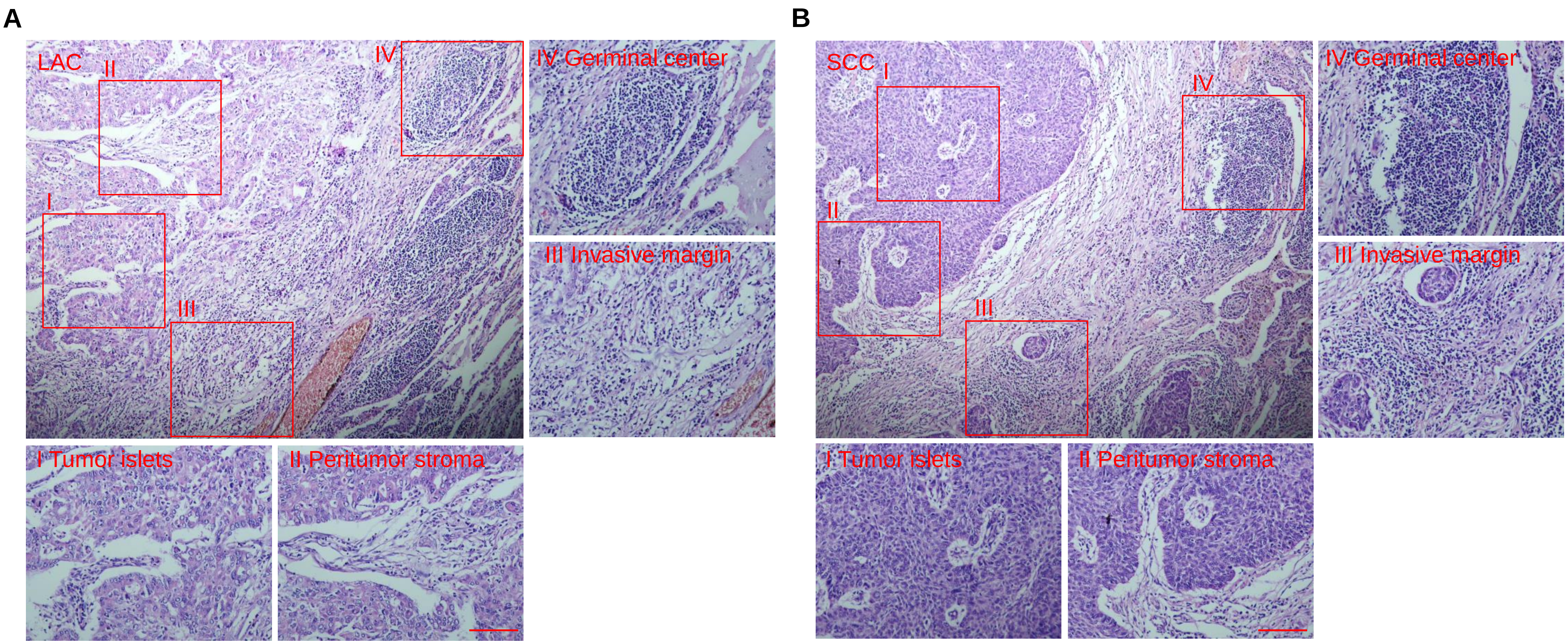

Supplementary Figure S1 Representative images of hematoxylin and eosin staining showed different tissue regions of tumor islets, peritumor stroma, invasive margin and germinal center in LAC (A) and SCC (B) . Scale bar, 50 μm.

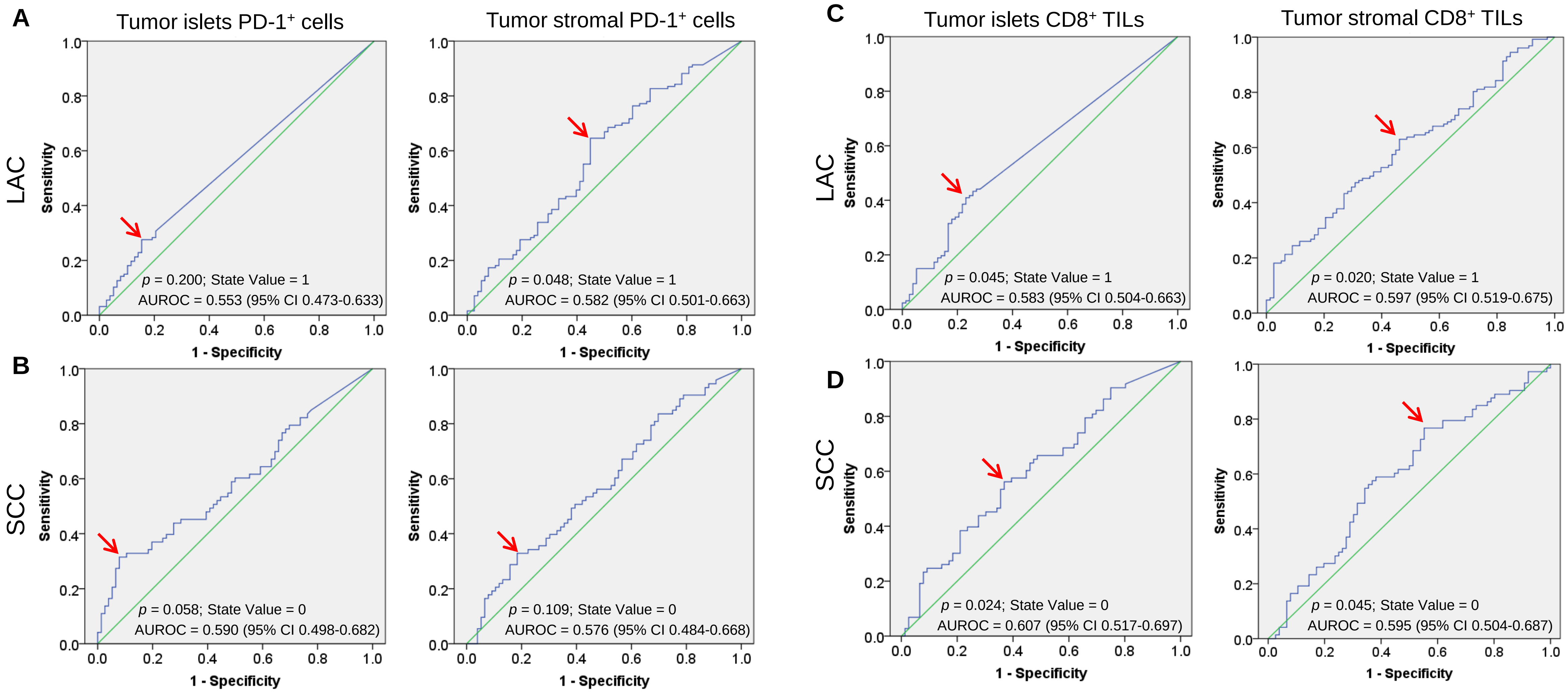

Supplementary Figure S2 The cut-off value of TILs by ROC curve analysis. For PD-1$^+$ cells in the tumor islet and stroma, (A) 14 cells/mm^2 and 217 cells/mm^2 were used as the cut-off value by ROC curve analysis in LAC, respectively; (B)The cut-off value of 122 cells/mm^2 and 664 cells/mm^2 was used in SCC, respectively. For CD8$^+$ T cells in the tumor islet and stroma (C and D), 25 cells/mm^2 and 710 cells/mm^2 were regarded as the cut-off value in LAC and 111 cells/mm^2 and 734 cells/mm^2 were regarded as the cut-off value in SCC, respectively.

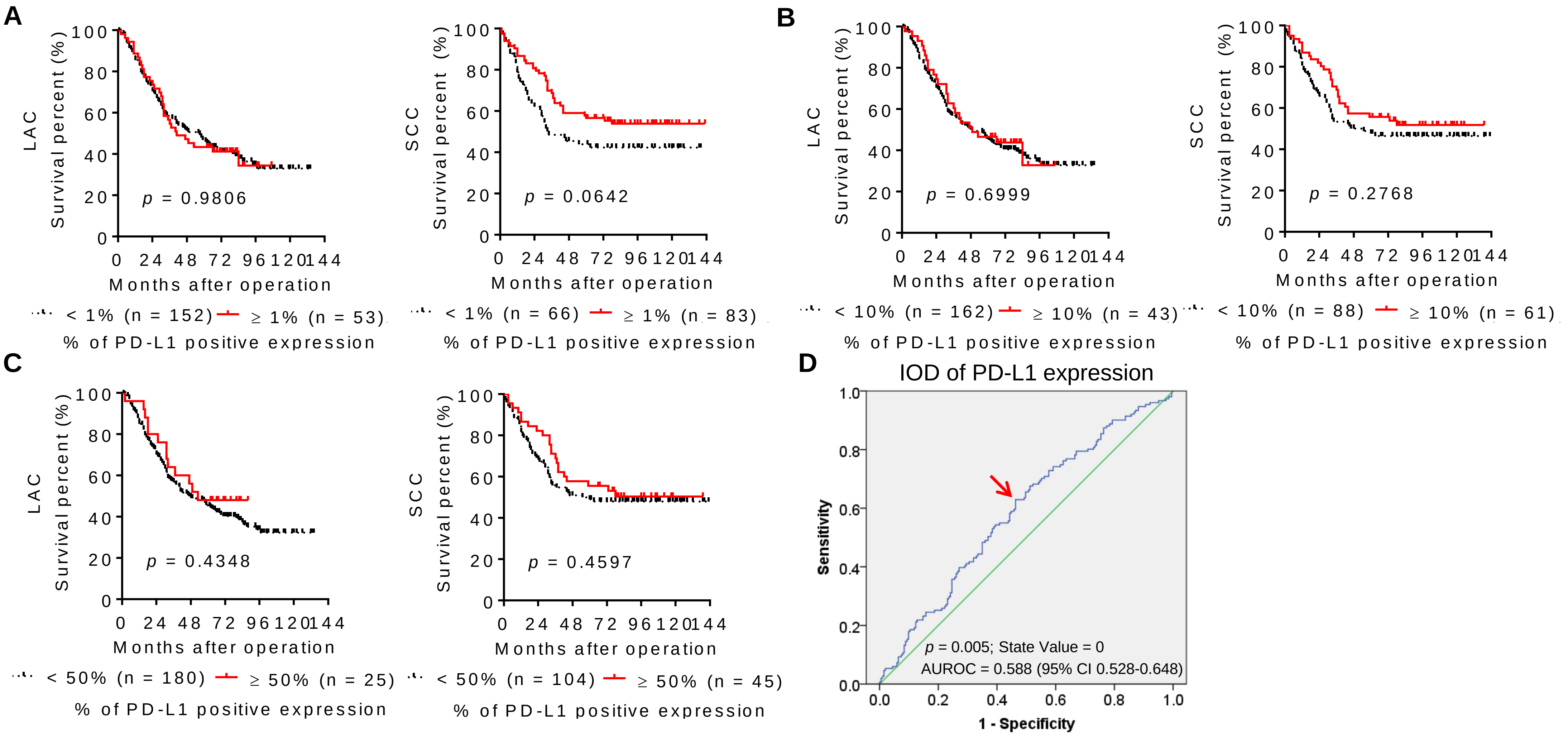

Supplementary Figure S3 Kaplan-Meier survival curves for PD-L1 expression in LAC and SCC (A-C). PD-L1 expression was not significantly associated with survival of both LAC and SCC patients, regardless of the cut-off value of ≥1%, ≥10% or ≥50% for positivity, respectively. *P*-values were calculated using the log-rank test. (D) The cut-off value of tumor cell PD-L1 expression in LAC and SCC was 0.655 IOD/10^4 by ROC curve analysis.

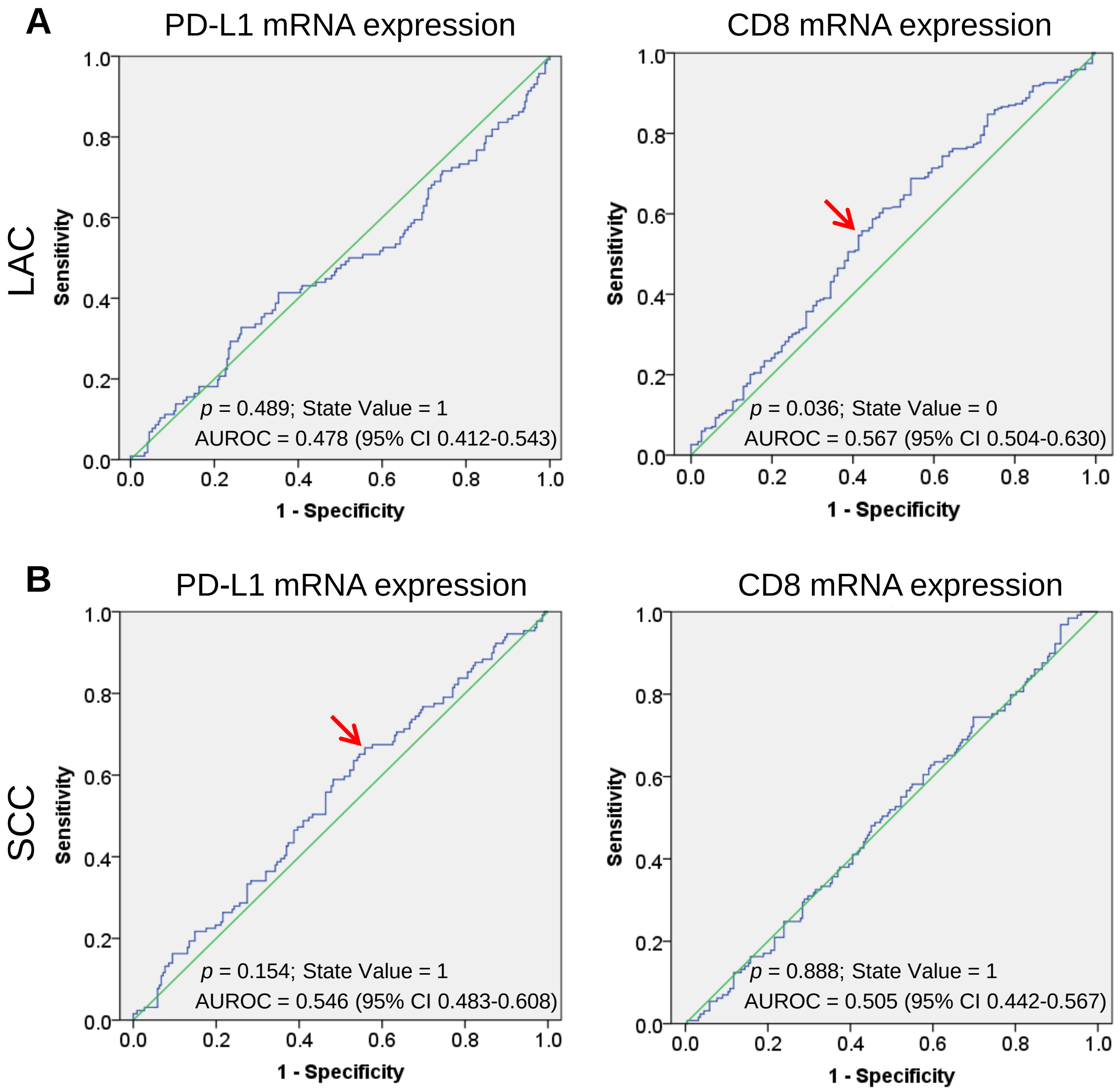

Supplementary Figure S4 The cut-off value of TCGA data by ROC curve analysis. The ROC curves for the mRNA expression of PD-L1 in LAC and for the mRNA expression of CD8 intersected the reference line above 0.2, so the median was selected for the cut-off value. (A) The median 1.118 was used for the cut-off value of PD-L1 expression and 1.071 based on ROC curve was used for the cur-off value of CD8 expression in 385 LAC. (B) 1.105 based on ROC curve was used for the cur-off value of PD-L1 expression and the median 0.907 was used for the cut-off value of CD8 expression and in 351 SCC.
