# Supplementary material for: The landscape of immune microenvironment in lung adenocarcinoma and squamous cell carcinoma based on PD‐L1 expression and tumor‐infiltrating lymphocytes
Source: Cancer Med. 2019 Oct 11;8(17):7207–18. doi: 10.1002/cam4.2580 (PMC6885882; doi:10.1002/cam4.2580)
Supplement: Supplementary file 2 [file CAM4-8-7207-s002.docx]

**Supplementary Table S1.** Clinical characteristics associated with the distribution of PD-1 and CD8 TILs in lung adenocarcinoma and squamous cell carcinoma

| Clinical | PD-1 | | | | | | | | | | | | | |  | | | CD8 | | | | | | | | | | | | | | |
| --- | --- | --- | --- | --- | --- | --- | --- | --- | --- | --- | --- | --- | --- | --- | --- | --- | --- | --- | --- | --- | --- | --- | --- | --- | --- | --- | --- | --- | --- | --- | --- | --- |
|  | Tumor islets | | | | | | | Tumor stromal | | | | | | |  | | | Tumor islets | | | | | | | Tumor stromal | | | | | | | |
| characteristic | Low  n (%) | | | High  n (%) | | *P* | | | Low  n (%) | | | High  n (%) | | *P* | | | Low  n (%) | | | | High  n (%) | | *P* | | | Low  n (%) | | | High  n (%) | | *P* | |
| **LAC (n = 205)** | | |  | |  | |  | | | |  | |  | | | |  | | |  | |  | | | |  | |  | |  | | |
| Gender |  | | | 0.177 | | | | |  | | | 0.065 | | | | |  | | | | 0.372 | | | | |  | | | 0.088 | | | |
| Female | 70 (82) | | | 15 (18) | |  | | | 43 (51) | | | 42 (49) | |  | | | 59 (69) | | | | 26 (31) | |  | | | 43 (51) | | | 42 (49) | |  | |
| Male | 88 (73) | | | 32 (27) | |  | | | 45 (38) | | | 75 (62) | |  | | | 75 (63) | | | | 45 (37) | |  | | | 46 (38) | | | 74 (62) | |  | |
| Age (years) |  | | | 0.868 | | | | |  | | | 0.395 | | | | |  | | | | 0.238 | | | | |  | | | 0.088 | | | |
| ≤60 | 68 (76) | | | 21 (24) | |  | | | 35 (39) | | | 54 (61) | |  | | | 54 (61) | | | | 35 (39) | |  | | | 45 (51) | | | 44 (49) | |  | |
| >60 | 90 (77) | | | 26 (23) | |  | | | 53 (46) | | | 63 (54) | |  | | | 80 (69) | | | | 36 (31) | |  | | | 44 (38) | | | 72 (62) | |  | |
| Smoking |  | | | 0.132 | | | | |  | | | 0.674 | | | | |  | | | | 0.557 | | | | |  | | | 0.321 | | | |
| No | 92 (81) | | | 21 (19) | |  | | | 47 (42) | | | 66 (58) | |  | | | 76 (67) | | | | 37 (33) | |  | | | 53 (47) | | | 60 (53) | |  | |
| Yes | 66 (72) | | | 26 (28) | |  | | | 41 (45) | | | 51 (55) | |  | | | 58 (63) | | | | 34 (37) | |  | | | 36 (39) | | | 56 (61) | |  | |
| Tumor size |  | | | 0.088 | | | | |  | | | 0.314 | | | | |  | | | | 0.653 | | | | |  | | | 0.566 | | | |
| ≤3cm | 101 (81) | | | 23 (19) | |  | | | 57 (46) | | | 67 (54) | |  | | | 83 (67) | | | | 41 (33) | |  | | | 56 (45) | | | 68 (55) | |  | |
| >3cm | 57 (70) | | | 24 (30) | |  | | | 31 (38) | | | 50 (62) | |  | | | 51 (63) | | | | 30 (37) | |  | | | 33 (41) | | | 48 (59) | |  | |
| Lymph node metastasis | | | | 0.384 | | | | |  | | | 0.553 | | | | |  | | | | 0.123 | | | | |  | | | 0.883 | | | |
| No | 106 (79) | | | 28 (21) | |  | | | 60 (45) | | | 74 (55) | |  | | | 93 (69) | | | | 41 (31) | |  | | | 59 (44) | | | 75 (56) | |  | |
| Yes | 52 (73) | | | 19 (27) | |  | | | 28 (39) | | | 43 (61) | |  | | | 41 (58) | | | | 30 (42) | |  | | | 30 (42) | | | 41 (58) | |  | |
| Stage |  | | | 0.081 | | | | |  | | | 0.371 | | | | |  | | | | 0.102 | | | | |  | | | 0.205 | | | |
| I | 91 (83) | | | 19 (17) | |  | | | 52 (47) | | | 58 (53) | |  | | | 79 (72) | | | | 31 (28) | |  | | | 54 (49) | | | 56 (51) | |  | |
| II | 30 (67) | | | 15 (33) | |  | | | 18 (40) | | | 27 (60) | |  | | | 25 (56) | | | | 20 (44) | |  | | | 16 (36) | | | 29 (64) | |  | |
| III and IV | 37 (74) | | | 13 (26) | |  | | | 18 (36) | | | 32 (64) | |  | | | 30 (60) | | | | 20 (40) | |  | | | 19 (38) | | | 31 (62) | |  | |
| Differentiation |  | | | 0.0001 | | | | |  | | | 0.723 | | | | |  | | | | 0.004 | | | | |  | | | 0.401 | | | |
| Well | 42 (95) | | | 2 (5) | |  | | | 20 (45) | | | 24 (55) | |  | | | 35 (80) | | | | 9 (20) | |  | | | 20 (45) | | | 24 (55) | |  | |
| Moderate | 90 (78) | | | 26 (22) | |  | | | 47 (40) | | | 69 (60) | |  | | | 78 (67) | | | | 38 (33) | |  | | | 46 (40) | | | 70 (60) | |  | |
| Poor | 26 (56) | | | 19 (42) | |  | | | 21 (47) | | | 24 (53) | |  | | | 21 (47) | | | | 24 (53) | |  | | | 23 (51) | | | 22 (49) | |  | |
| Recurrence within 5 years | | | | 0.218 | | | | |  | | | 0.667 | | | | |  | | | | 1.000 | | | | |  | | | 0.007 | | | |
| No | 111 (74) | | | 39 (26) | |  | | | 66 (44) | | | 84 (56) | |  | | | 98 (65) | | | | 52 (35) | |  | | | 74 (49) | | | 76 (51) | |  | |
| Yes | 47 (85) | | | 8 (15) | |  | | | 22 (40) | | | 33 (60) | |  | | | 36 (65) | | | | 19 (35) | |  | | | 15 (27) | | | 40 (73) | |  | |
| Histology subtype | | | | 0.0001 | | | | |  | | | 0.241 | | | | |  | | | | 0.005 | | | | |  | | | 0.948 | | | |
| Lepidic | 28 (93) | | | 2 (7) | |  | | | 11 (37) | | | 19 (53) | |  | | | 26 (87) | | | | 4 (13) | |  | | | 13 (43) | | | 17 (57) | |  | |
| Acinar | 98 (80) | | | 25 (20) | |  | | | 49 (40) | | | 74 (60) | |  | | | 82 (67) | | | | 41 (33) | |  | | | 52 (42) | | | 71 (58) | |  | |
| Papillary | 15 (94) | | | 1 (6) | |  | | | 10 (62) | | | 6 (38) | |  | | | 10 (63) | | | | 6 (37) | |  | | | 8 (50) | | | 8 (50) | |  | |
| Solid | 17 (47) | | | 19 (53) | |  | | | 18 (50) | | | 18 (50) | |  | | | 16 (44) | | | | 20 (56) | |  | | | 16 (44) | | | 20 (56) | |  | |
| **SCC (n = 149)** | | |  | |  | |  | | | |  | |  | | |  | | | |  | |  | |  | | | |  | |  | |  |
| Gender |  | | | 1.000 | | | | |  | | | 1.000 | | | | |  | | | | 1.000 | | | | |  | | | 1.000 | | | |
| Female | 4 (100) | | | 0 (0) | |  | | | 4 (100) | | | 0 (0) | |  | | | 2 (50) | | | | 2 (50) | |  | | | 1 (25) | | | 3 (75) | |  | |
| Male | 116 (80) | | | 29 (20) | |  | | | 107 (74) | | | 38 (26) | |  | | | 78 (54) | | | | 67 (46) | |  | | | 50 (34) | | | 95 (66) | |  | |
| Age (years) |  | | | 0.680 | | | | |  | | | 0.259 | | | | |  | | | | 0.508 | | | | |  | | | 0.121 | | | |
| ≤60 | 52 (79) | | | 14 (21) | |  | | | 46 (70) | | | 20 (30) | |  | | | 33 (50) | | | | 33 (50) | |  | | | 18 (27) | | | 48 (73) | |  | |
| >60 | 68 (82) | | | 15 (18) | |  | | | 65 (78) | | | 18 (22) | |  | | | 47 (57) | | | | 36 (43) | |  | | | 33 (40) | | | 50 (60) | |  | |
| Smoking |  | | | 0.336 | | | | |  | | | 1.000 | | | | |  | | | | 0.092 | | | | |  | | | 0.615 | | | |
| No | 18 (90) | | | 2 (10) | |  | | | 15 (75) | | | 5 (25) | |  | | | 7 (35) | | | | 13 (65) | |  | | | 8 (40) | | | 12 (60) | |  | |
| Yes | 102 (79) | | | 27 (21) | |  | | | 96 (74) | | | 33 (26) | |  | | | 73 (57) | | | | 56 (43) | |  | | | 43 (33) | | | 86 (67) | |  | |
| Tumor size |  | | | 0.818 | | | | |  | | | 0.831 | | | | |  | | | | 0.014 | | | | |  | | | 0.434 | | | |
| ≤3cm | 31 (79) | | | 8 (21) | |  | | | 30 (77) | | | 9 (23) | |  | | | 14 (36) | | | | 25 (64) | |  | | | 11 (28) | | | 28 (72) | |  | |
| >3cm | 89 (81) | | | 21 (19) | |  | | | 81 (74) | | | 29 (26) | |  | | | 66 (60) | | | | 44 (40) | |  | | | 40 (36) | | | 70 (46) | |  | |
| Lymph node metastasis | | | | 0.660 | | | | |  | | | 1.000 | | | | |  | | | | 0.727 | | | | |  | | | 0.712 | | | |
| No | 80 (79) | | | 21 (21) | |  | | | 75 (74) | | | 26 (26) | |  | | | 53 (52) | | | | 48 (48) | |  | | | 36 (36) | | | 65 (64) | |  | |
| Yes | 40 (83) | | | 8 (17) | |  | | | 36 (75) | | | 12 (25) | |  | | | 27 (56) | | | | 21 (44) | |  | | | 15 (31) | | | 33 (69) | |  | |
| Stage |  | | | 0.303 | | | | |  | | | 0.865 | | | | |  | | | | 0.128 | | | | |  | | | 0.644 | | | |
| I | 54 (77) | | | 16 (23) | |  | | | 53 (76) | | | 17 (24) | |  | | | 33 (47) | | | | 37 (53) | |  | | | 25 (36) | | | 45 (64) | |  | |
| II | 33 (79) | | | 9 (21) | |  | | | 30 (71) | | | 12 (29) | |  | | | 22 (52) | | | | 20 (48) | |  | | | 12 (29) | | | 30 (71) | |  | |
| III and IV | 33 (89) | | | 4 (11) | |  | | | 28 (76) | | | 9 (24) | |  | | | 25 (68) | | | | 12 (32) | |  | | | 14 (38) | | | 23 (62) | |  | |
| Differentiation |  | | | 0.808 | | | | |  | | | 0.952 | | | | |  | | | | 0.903 | | | | |  | | | 0.342 | | | |
| Well | 10 (83) | | | 2 (17) | |  | | | 9 (75) | | | 3 (25) | |  | | | 7 (58) | | | | 5 (42) | |  | | | 3 (25) | | | 9 (75) | |  | |
| Moderate | 91 (81) | | | 21 (19) | |  | | | 84 (75) | | | 28 (25) | |  | | | 59 (53) | | | | 53 (47) | |  | | | 42 (38) | | | 70 (42) | |  | |
| Poor | 19 (76) | | | 6 (24) | |  | | | 18 (72) | | | 7 (28) | |  | | | 14 (56) | | | | 11 (44) | |  | | | 6 (24) | | | 19 (76) | |  | |
| Recurrence within 5 years | | 0.107 | | | | | | |  | 0.809 | | | | | | |  | | 0.200 | | | | | | |  | 0.263 | | | | | |
| No | 95 (78) | | | 27 (22) | |  | | | 90 (62) | | | 32 (38) | |  | | | 62 (51) | | | | 60 (49) | |  | | | 39 (32) | | | 83 (68) | |  | |
| Yes | 25 (93) | | | 2 (7) | |  | | | 21 (78) | | | 6 (22) | |  | | | 18 (67) | | | | 9 (33) | |  | | | 12 (44) | | | 15 (56) | |  | |

Abbreviations: LAC, lung adenocarcinoma; PD-1, programmed cell death 1; SCC, squamous cell carcinoma; TIL, tumor-infiltrating lymphocytes

**Supplementary Table S2.** Clinical characteristics associated with tumor cell and immune cell PD-L1 expression in lung adenocarcinoma and squamous cell carcinoma

| Clinical | Tumor cell PD-L1 expression | | | |  | | Immune cell PD-L1 expression | | |
| --- | --- | --- | --- | --- | --- | --- | --- | --- | --- |
| characteristic | Low  n (%) | High  n (%) | | *P* |  | | Absent  n (%) | Present  n (%) | *P* |
| **LAC (n = 205)** | |  | | |  | |  |  | |
| Gender |  |  | 0.392 | | |  |  | 0.389 | |
| Female | 51 (60) | 34 (40) |  | | |  | 32 (38) | 53 (62) |  |
| Male | 64 (53) | 56 (47) |  | | |  | 53 (44) | 67 (56) |  |
| Age (years) |  |  | 0.159 | | |  |  | 0.394 | |
| ≤60 | 55 (62) | 34 (38) |  | | |  | 40 (45) | 49 (55) |  |
| >60 | 60 (52) | 56 (48) |  | | |  | 45 (39) | 71 (61) |  |
| Smoking |  |  | 0.888 | | |  |  | 0.064 | |
| No | 64 (57) | 49 (43) |  | | |  | 40 (35) | 73 (65) |  |
| Yes | 51 (55) | 41 (45) |  | | |  | 45 (49) | 47 (51) |  |
| Tumor size |  |  | 1.000 | | |  |  | 0.772 | |
| ≤3cm | 70 (56) | 54 (44) |  | | |  | 50 (40) | 74 (60) |  |
| >3cm | 45 (56) | 36 (44) |  | | |  | 35 (43) | 46 (57) |  |
| Lymph node metastasis | |  | 0.337 | | |  |  |  | 0.551 |
| No | 72 (54) | 62 (46) |  | | |  | 58 (43) | 76 (57) |  |
| Yes | 43 (60) | 28 (40) |  | | |  | 27 (38) | 44 (62) |  |
| Stage |  |  | 0.404 | | |  |  | 0.277 | |
| I | 57 (52) | 53 (48) |  | | |  | 48 (44) | 62 (56) |  |
| II | 28 (62) | 17 (38) |  | | |  | 21 (47) | 24 (53) |  |
| III and IV | 30 (60) | 20 (40) |  | | |  | 16 (32) | 34 (68) |  |
| Differentiation | |  | 0.704 | | |  |  | 0.638 | |
| Well | 23 (52) | 21 (48) |  | | |  | 19 (43) | 25 (57) |  |
| Moderate | 68 (59) | 48 (41) |  | | |  | 45 (39) | 71 (61) |  |
| Poor | 24 (53) | 21 (47) |  | | |  | 21 (47) | 24 (53) |  |
| Recurrence within 5 years | |  | 0.428 | | |  |  | 1.000 | |
| No | 87 (58) | 63 (42) |  | | |  | 62 (41) | 88 (59) |  |
| Yes | 28 (51) | 27 (49) |  | | |  | 23 (42) | 32 (58) |  |
| Histology subtype | |  | 0.494 | | |  |  | 0.891 | |
| Lepidic | 15 (50) | 15 (50) |  | | |  | 12 (40) | 18 (60) |  |
| Acinar | 73 (59) | 50 (41) |  | | |  | 53 (43) | 70 (57) |  |
| Papillary | 10 (62) | 6 (38) |  | | |  | 7 (44) | 9 (56) |  |
| Solid | 17 (47) | 19 (53) |  | | |  | 13 (36) | 23 (64) |  |
| **SCC (n = 149)** | |  |  | | |  |  |  |  |
| Gender |  |  | 0.301 | | |  |  | 0.573 | |
| Female | 0 (0) | 4 (100) |  | | |  | 0 (0) | 4 (100) |  |
| Male | 50 (34) | 95 (66) |  | | |  | 35 (24) | 110 (76) |  |
| Age (years) |  |  | 0.165 | | |  |  | 0.697 | |
| ≤60 | 18 (27) | 48 (73) |  | | |  | 14 (21) | 52 (79) |  |
| >60 | 32 (39) | 51 (61) |  | | |  | 21 (25) | 62 (75) |  |
| Smoking |  |  | 0.454 | | |  |  | 0.162 | |
| No | 5 (25) | 15 (75) |  | | |  | 2 (10) | 18 (90) |  |
| Yes | 45 (35) | 84 (65) |  | | |  | 33 (26) | 96 (74) |  |
| Tumor size |  |  | 0.438 | | |  |  | 0.826 | |
| ≤3cm | 11 (28) | 28 (72) |  | | |  | 10 (26) | 29 (74) |  |
| >3cm | 39 (35) | 71 (65) |  | | |  | 25 (23) | 85 (77) |  |
| Lymph node metastasis | |  | 1.000 | | |  |  | 0.682 | |
| No | 34 (34) | 67 (66) |  | | |  | 25 (25) | 76 (75) |  |
| Yes | 16 (33) | 32 (67) |  | | |  | 10 (21) | 38 (79) |  |
| Stage |  |  | 0.166 | | |  |  | 0.033 | |
| I | 20 (29) | 50 (71) |  | | |  | 16 (23) | 54 (77) |  |
| II | 19 (45) | 23 (55) |  | | |  | 15 (36) | 27 (64) |  |
| III and IV | 11 (30) | 26 (70) |  | | |  | 4 (11) | 33 (89) |  |
| Differentiation | |  | 0.614 | | |  |  | 0.844 | |
| Well | 3 (25) | 9 (75) |  | | |  | 2 (17) | 10 (83) |  |
| Moderate | 40 (36) | 72 (64) |  | | |  | 27 (24) | 85 (76) |  |
| Poor | 7 (28) | 18 (72) |  | | |  | 6 (24) | 19 (76) |  |
| Recurrence within 5 years | |  | 0.113 | | |  |  |  | 0.453 |
| No | 37 (30) | 85 (70) |  | | |  | 27 (22) | 95 (78) |  |
| Yes | 13 (48) | 14 (52) |  | | |  | 8 (30) | 19 (70) |  |

Abbreviations: LAC, lung adenocarcinoma; PD-L1, programmed cell death ligand 1; SCC, squamous cell carcinoma

**Supplementary Table S3** Significant independent prognostic factors by Cox-regression analysis from TCGA database

| Factor | Univariate Cox-regression | |  | Multivariate Cox-regression ^a^ | |
| --- | --- | --- | --- | --- | --- |
|  | HR (95% CI) | *p* |  | HR (95% CI) | *p* |
| **LAC (n = 385)** |  |  |  |  |  |
| Gender | 0.948 (0.656-1.369) | 0.775 |  |  |  |
| Age | 1.003 (0.983-1.024) | 0.746 |  |  |  |
| Smoking | 0.863 (0.596-1.251) | 0.437 |  |  |  |
| Stage | 1.668 (1.407-1.977) | <0.001 |  | 1.668 (1.407-1.977) | <0.001 |
| Histology subtype | 1.026 (0.884-1.191) | 0.733 |  |  |  |
| RNA expression of PD-L1 | 1.010 (0.906-1.127) | 0.853 |  |  |  |
| RNA expression of PD-1 | 1.024 (0.900-1.164) | 0.722 |  |  |  |
| RNA expression of CD8A | 0.914 (0.805-1.037) | 0.163 |  |  |  |
| TMIT (PD-L1 and CD8A) |  |  |  |  |  |
| TMIT I | 1.000 |  |  |  |  |
| TMIT II | 1.067 (0.686-1.658) | 0.775 |  |  |  |
| TMIT III | 1.192 (0.691-2.055) | 0.529 |  |  |  |
| TMIT IV | 0.860 (0.454-1.627) | 0.642 |  |  |  |
| **SCC (n = 351)** |  |  |  |  |  |
| Gender | 1.324 (0.869-2.017) | 0.191 |  |  |  |
| Age | 1.023 (1.000-1.045) | 0.047 |  |  |  |
| Smoking | 0.958 (0.669-1.371) | 0.813 |  |  |  |
| Stage | 1.223 (0.996-1.501) | 0.054 |  |  |  |
| RNA expression of PD-L1 | 1.018 (0.923-1.123) | 0.722 |  |  |  |
| RNA expression of PD-1 | 1.058 (0.945-1.186) | 0.328 |  |  |  |
| RNA expression of CD8A | 1.001 (0.891-1.124) | 0.987 |  |  |  |
| TMIT (PD-L1 and CD8A) |  |  |  |  |  |
| TMIT I | 1.000 |  |  |  |  |
| TMIT II | 0.842 (0.536-1.322) | 0.455 |  |  |  |
| TMIT III | 0.909 (0.587-1.408) | 0.669 |  |  |  |
| TMIT IV | 0.832 (0.452-1.530) | 0.553 |  |  |  |

^a^ Only statistically signiﬁcant variables obtained from the univariate model were included in the multivariate analysis. Results of multivariate analysis showing signiﬁcant independent prognostic factors.

Abbreviations: CI, conﬁdence interval; HR, hazard ratio; LAC, lung adenocarcinoma; PD-1, programmed cell death 1; PD-L1, programmed cell death ligand 1; SCC, squamous cell carcinoma; TCGA, the cancer genome atlas; TMIT, tumor microenvironment immune types; TMIT I, PD-L1^high^ and CD8A^high^; TMIT II , PD-L1^low^ and CD8A^low^; TMIT III, PD-L1^high^ and CD8A^low^; TMIT IV, PD-L1^low^ and CD8A^high^

**Supplementary Table S4.** Clinical characteristics associated with TMIT in lung adenocarcinoma and squamous cell carcinoma

| Clinical | TMIT (PD-L1 and TIL tumor islets) | | | | | | | | | | | | | | | |  | | TMIT (PD-L1 and TIL tumor stromal) | | | | | | | | | | | |  | | |
| --- | --- | --- | --- | --- | --- | --- | --- | --- | --- | --- | --- | --- | --- | --- | --- | --- | --- | --- | --- | --- | --- | --- | --- | --- | --- | --- | --- | --- | --- | --- | --- | --- | --- |
| characteristic | I  n (%) | | II  n (%) | | | III  n (%) | | | | | | IV  n (%) | | | | | *P* | | I  n (%) | II  n (%) | | | | | III  n (%) | | | | IV  n (%) | | | | *P* |
| **LAC (n = 205)** |  | |  | | |  | | | | | |  | | | | |  | |  |  | | | | |  | | | |  | | | |  |
| Gender |  | |  | | | | | | | |  | | | | | 0.141 | | |  | | | |  | | | |  | | 0.239 | | | | |
| Female | 6 (6) | | 30 (35) | | | 29 (34) | | | | | | 21 (25) | | | | |  | | 30 (24) | 29 (34) | | | | | 14 (16) | | | | 22 (26) | | | |  |
| Male | 20 (17) | | 39 (32) | | | 36 (30) | | | | | | 25 (21) | | | | |  | | 36 (30) | 26 (22) | | | | | 20 (17) | | | | 38 (31) | | | |  |
| Age (years) |  | |  | | | | | | | |  | | | | | 0.296 | | |  | | | |  | | | | 0.178 | | | | | | |
| ≤60 | 12 (13) | | 32 (36) | | | 22 (25) | | | | | | 23 (26) | | | | |  | | 19 (21) | 30 (34) | | | | | 15 (17) | | | | 25 (28) | | | |  |
| >60 | 13 (11) | | 37 (32) | | | 43 (37) | | | | | | 23 (20) | | | | |  | | 37 (32) | 25 (22) | | | | | 19 (16) | | | | 35 (30) | | | |  |
| Smoking |  | |  | | | | | | | |  | | | | | 0.356 | | |  | | | |  | | | | 0.424 | | | | | | |
| No | 10 (9) | | 37 (33) | | | 39 (34) | | | | | | 27 (24) | | | | |  | | 31 (27) | 35 (31) | | | | | 18 (16) | | | | 29 (26) | | | |  |
| Yes | 15 (16) | | 32 (35) | | | 26 (28) | | | | | | 19 (21) | | | | |  | | 25 (27) | 20 (22) | | | | | 16 (17) | | | | 31 (34) | | | |  |
| Tumor size |  | |  | | | | | | | |  | | | | | 0.940 | | |  | | | |  | | | | 0.935 | | | | | | |
| ≤3cm | 14 (11) | | 43 (35) | | | 40 (32) | | | | | | 27 (22) | | | | |  | | 33 (27) | 35 (28) | | | | | 21 (17) | | | | 35 (28) | | | |  |
| >3cm | 11 (13) | | 26 (32) | | | 25 (31) | | | | | | 19 (24) | | | | |  | | 23 (28) | 20 (25) | | | | | 13 (16) | | | | 25 (31) | | | |  |
| Lymph node metastasis | | | |  | | | |  | | | | | 0.268 | | | | | |  |  | | | | 0.460 | | | | | | | | | |
| No | 14 (10) | | 45 (34) | | | 48 (36) | | | | | | 27 (20) | | | | |  | | 36 (27) | 33 (25) | | | | | 26 (19) | | | | 39 (29) | | | |  |
| Yes | 11 (15) | | 24 (34) | | | 17 (24) | | | | | | 19 (27) | | | | |  | | 20 (28) | 22 (31) | | | | | 8 (11) | | | | 21 (30) | | | |  |
| Stage |  | |  | | | | | | | |  | | | | | 0.128 | | |  | | | |  | | | | 0.169 | | | | | | |
| I | 9 (8) | | 35 (22) | | | 44 (40) | | | | | | 22 (20) | | | | |  | | 27 (25) | 28 (25) | | | | | 26 (24) | | | | 29 (26) | | | |  |
| II | 7 (16) | | 15 (33) | | | 10 (22) | | | | | | 13 (29) | | | | |  | | 13 (29) | 12 (27) | | | | | 4 (9) | | | | 16 (35) | | | |  |
| III and IV | 9 (18) | | 19 (38) | | | 11 (22) | | | | | | 11 (22) | | | | |  | | 16 (32) | 15 (30) | | | | | 4 (8) | | | | 15 (30) | | | |  |
| Differentiation | |  | |  | | | |  | | | | | 0.027 | | | | | |  |  | | | | 0.246 | | | | | | | | | |
| Well | 2 (5) | | 16 (36) | | | 19 (43) | | | | | | 7 (16) | | | | |  | | 14 (32) | 13 (29) | | | | | 7 (16) | | | | 10 (23) | | | |  |
| Moderate | 12 (10) | | 42 (36) | | | 36 (31) | | | | | | 26 (23) | | | | |  | | 28 (24) | 26 (23) | | | | | 20 (17) | | | | 42 (36) | | | |  |
| Poor | 11 (24) | | 11 (24) | | | 10 (23) | | | | | | 13 (29) | | | | |  | | 14 (31) | 16 (35) | | | | | 7 (16) | | | | 8 (18) | | | |  |
| Recurrence with 5 years | | | | |  | | | | |  | | | | | 0.798 | | | |  | | |  | | | | 0.026 | | | | | | | |
| No | 18 (12) | | 53 (35) | | | 45 (30) | | | | | | 34 (23) | | | | |  | | 37 (25) | 48 (32) | | | | | 26 (17) | | | | 39 (26) | | | |  |
| Yes | 7 (13) | | 16 (29) | | | 20 (36) | | | | | | 12 (22) | | | | |  | | 19 (34) | 7 (13) | | | | | 8 (15) | | | | 21 (38) | | | |  |
| Histology subtype | |  | |  | | | |  | | | | | 0.036 | | | | | |  |  | | | | 0.945 | | | | | | | | | |
| Lepidic | 0 (0) | | 11 (37) | | | 15 (50) | | | | | | 4 (13) | | | | |  | | 10 (33) | 8 (27) | | | | | 5 (17) | | | | 7 (23) | | | |  |
| Acinar | 14 (11) | | 36 (29) | | | 36 (29) | | | | | | 27 (21) | | | | |  | | 31 (25) | 33 (27) | | | | | 19 (15) | | | | 40 (33) | | | |  |
| Papillary | 1 (7) | | 5 (31) | | | 5 (31) | | | | | | 5 (31) | | | | |  | | 3 (19) | 5 (31) | | | | | 3 (19) | | | | 5 (31) | | | |  |
| Solid | 10 (28) | | 7 (19) | | | 9 (25) | | | | | | 10 (28) | | | | |  | | 12 (33) | 9 (25) | | | | | 7 (19) | | | | 8 (23) | | | |  |
| **SCC (n = 149)** | |  | |  | | | | |  | | | | |  | | | |  |  | |  | | | | | | |  | |  | |  | |
| Gender |  | |  | | | | | | | |  | | | | | 0.553 | | |  | | | |  | | | | 0.540 | | | | | | |
| Female | 2 (50) | | 0 (0) | | | 2 (50) | | | | | | 0 (0) | | | | |  | | 3 (75) | 0 (0) | | | | | 1 (25) | | | | 0 (0) | | | |  |
| Male | 116 (80) | | 29 (20) | | | 107 (74) | | | | | | 38 (26) | | | | |  | | 66 (46) | 21 (14) | | | | | 29 (20) | | | | 29 (20) | | | |  |
| Age (years) |  | |  | | | | | | | |  | | | | | 0.478 | | |  | | | |  | | | | 0.033 | | | | | | |
| ≤60 | 26 (39) | | 11 (17) | | | 22 (33) | | | | | | 7 (11) | | | | |  | | 39 (58) | 9 (14) | | | | | 9 (14) | | | | 9 (14) | | | |  |
| >60 | 26 (31) | | 22 (27) | | | 25 (30) | | | | | | 10 (12) | | | | |  | | 30 (36) | 12 (15) | | | | | 21 (25) | | | | 20 (24) | | | |  |
| Smoking |  | |  | | | | | | | |  | | | | | 0.305 | | |  | | | |  | | | | 0.329 | | | | | | |
| No | 10 (50) | | 2 (10) | | | 5 (25) | | | | | | 3 (15) | | | | |  | | 11 (55) | 4 (20) | | | | | 4 (20) | | | | 1 (5) | | | |  |
| Yes | 42 (33) | | 31 (24) | | | 42 (33) | | | | | | 14 (10) | | | | |  | | 58 (45) | 17 (13) | | | | | 26 (20) | | | | 28 (22) | | | |  |
| Tumor size |  | |  | | | | | | | |  | | | | | <0.0001 | | |  | | | |  | | | | 0.708 | | | | | | |
| ≤3cm | 19 (49) | | 5 (13) | | | 9 (23) | | | | | | 6 (15) | | | | |  | | 21 (44) | 4 (10) | | | | | 7 (18) | | | | 7 (18) | | | |  |
| >3cm | 33 (30) | | 28 (25) | | | 38 (35) | | | | | | 11 (10) | | | | |  | | 48 (44) | 17 (15) | | | | | 23 (21) | | | | 22 (20) | | | |  |
| Lymph node metastasis | | | |  | | | |  | | | | | 0.977 | | | | | |  |  | | | | 0.548 | | | | | | | | | |
| No | 36 (36) | | 22 (22) | | | 31 (30) | | | | | | 12 (12) | | | | |  | | 47 (46) | 17 (17) | | | | | 20 (20) | | | | 18 (17) | | | |  |
| Yes | 16 (33) | | 11 (23) | | | 16 (33) | | | | | | 5 (11) | | | | |  | | 22 (46) | 4 (8) | | | | | 10 (21) | | | | 11 (23) | | | |  |
| Stage |  | |  | | | | | | | |  | | | | | 0.108 | | |  | | | |  | | | | 0.465 | | | | | | |
| I | 28 (40) | | 11 (16) | | | 22 (31) | | | | | | 9 (13) | | | | |  | | 35 (50) | 10 (14) | | | | | 15 (22) | | | | 10 (14) | | | |  |
| II | 15 (36) | | 14 (33) | | | 8 (19) | | | | | | 5 (12) | | | | |  | | 17 (40) | 6 (15) | | | | | 6 (15) | | | | 13 (30) | | | |  |
| III and IV | 9 (24) | | 8 (22) | | | 17 46) | | | | | | 3 (8) | | | | |  | | 17 (46) | 5 (14) | | | | | 9 (24) | | | | 6 (16) | | | |  |
| Differentiation | |  | |  | | | |  | | | | | 0.681 | | | | | |  |  | | | | 0.827 | | | | | | | | | |
| Well | 5 (42) | | 3 (25) | | | 4 (33) | | | | | | 0 (0) | | | | |  | | 7 (58) | 1 (8) | | | | | 2 (17) | | | | 2 (17) | | | |  |
| Moderate | 37 (33) | | 24 (22) | | | 35 (31) | | | | | | 16 (14) | | | | |  | | 48 (43) | 18 (16) | | | | | 24 (22) | | | | 22 (19) | | | |  |
| Poor | 10 (40) | | 6 (24) | | | 8 (32) | | | | | | 1 (4) | | | | |  | | 14 (56) | 2 (8) | | | | | 4 (16) | | | | 5 (20) | | | |  |
| Recurrence within 5 years | | | | | | |  | | | | | | | | | 0.082 | | | 0.200 | | | | | | | | | | | | | | |
| No | 45 (37) | | 22 (18) | | | 40 (33) | | | | | | 15 (12) | | | | |  | | 61 (50) | 15 (12) | | | | | 24 (20) | | | | 22 (18) | | | |  |
| Yes | 7 (26) | | 11 (41) | | | 7 (26) | | | | | | 2 (7) | | | | |  | | 8 (30) | 6 (22) | | | | | 6 (22) | | | | 7 (26) | | | |  |

Abbreviations: LAC, lung adenocarcinoma; PD-L1, programmed cell death ligand 1; SCC, squamous cell carcinoma; TMIT, tumor microenvironment immune types, TMIT I, PD-L1^high^ and TIL^high^; TMIT II, PD-L1^low^ and TIL^low^; TMIT III, PD-L1^high^ and TIL^low^; TMIT IV, PD-L1^low^ and TIL^high^
